# Persistent changes in calcium-regulating hormones and bone turnover markers in living kidney donors more than 20 years after donation

**DOI:** 10.1093/jbmrpl/ziae067

**Published:** 2024-05-13

**Authors:** Brandon R Grossardt, Hilal Maradit Kremers, Adam R Miller, Bertram L Kasiske, Arthur J Matas, Sundeep Khosla, Walter K Kremers, Hatem Amer, Rajiv Kumar

**Affiliations:** Division of Clinical Trials and Biostatistics, Department of Quantitative Health Sciences, Mayo Clinic, Rochester, MN 55905, United States; Division of Epidemiology, Department of Quantitative Health Sciences, Mayo Clinic, Rochester, MN 55905, United States; Department of Orthopedics, Mayo Clinic, Rochester, MN 55905, United States; William J. von Liebig Center for Transplantation Clinical Research and Regeneration, Mayo Clinic, Rochester, MN 55905, United States; Division of Nephrology, Department of Medicine, Hennepin County Medical Center, Minneapolis, MN 55415, United States; Division of Transplantation, Department of Surgery, University of Minnesota, Minneapolis, MN 55455, United States; Division of Endocrinology, Diabetes and Metabolism, Department of Medicine and Kogod Center on Aging, Mayo Clinic, Rochester, MN 55905, United States; Department of Physiology and Biomedical Engineering, Mayo Clinic, Rochester, MN 55905, United States; Division of Clinical Trials and Biostatistics, Department of Quantitative Health Sciences, Mayo Clinic, Rochester, MN 55905, United States; William J. von Liebig Center for Transplantation Clinical Research and Regeneration, Mayo Clinic, Rochester, MN 55905, United States; Division of Nephrology and Hypertension, Department of Medicine, Mayo Clinic, Rochester, MN 55905, United States; Division of Nephrology and Hypertension, Department of Medicine, Mayo Clinic, Rochester, MN 55905, United States; Department of Biochemistry and Molecular Biology, Mayo Clinic, Rochester, MN 55905, United States

**Keywords:** living kidney donors, bone architecture, 1,25-dihydroxyvitamin D, parathyroid hormone, serum calcium

## Abstract

In a previous study, we observed decreased 1,25-dihydroxyvitamin D levels, secondary hyperparathyroidism, and increased bone turnover markers in living kidney donors (LKDs) at 3 months and 36 months after kidney donation. In our recent survey-based study, we found no increased risk of fractures of all types but observed significantly more vertebral fractures in LKDs compared with matched controls. To elucidate the long-term effects of kidney donation on bone health, we recruited 139 LKDs and 139 age and sex matched controls from the survey-based participants for further mechanistic analyses. Specifically, we assessed whether LKDs had persistent abnormalities in calcium- and phosphorus-regulating hormones and related factors, in bone formation and resorption markers, and in density and microstructure of bone compared with controls. We measured serum markers, bone mineral density (BMD), bone microstructure and strength (via high-resolution peripheral quantitative computed tomography and micro-finite element analysis [HRpQCT]), and advanced glycation end-products in donors and controls. LKDs had decreased 1,25-dihydroxyvitamin D concentrations (donors mean 33.89 pg/mL vs. controls 38.79 pg/mL, percent difference = -12.6%; *P* < .001), increases in both parathyroid hormone (when corrected for ionized calcium; donors mean 52.98 pg/mL vs. controls 46.89 pg/mL,% difference 13%; *P* = .03) and ionized calcium levels (donors mean 5.13 mg/dL vs. controls 5.04 mg/dL; *P* < .001), and increases in several bone resorption and formation markers versus controls. LKDs and controls had similar measures of BMD; however, HRpQCT suggested that LKDs have a statistically insignificant tendency toward thinner cortical bone and lower failure loads as measured by micro-finite element analysis. Our findings suggest that changes in the hormonal mileu after kidney donation and the long-term cumulative effects of these changes on bone health persist for decades after kidney donation and may explain later-life increased rates of vertebral fractures.

## Graphical Abstract

**Graphical Abstract:**
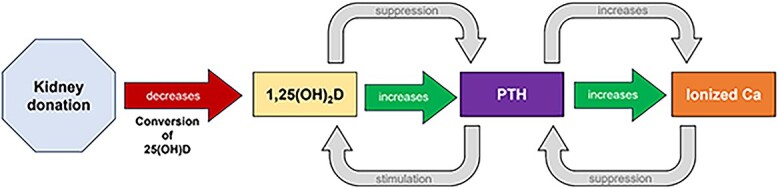

## Introduction

We previously demonstrated that living kidney donors (LKDs) had reduced glomerular filtration rate, decreased serum 1,25-dihydroxyvitamin D concentrations, increased parathyroid hormone (PTH; secondary hyperparathyroidism), and increased bone formation and resorption markers at 3 months and 36 months after kidney donation.^1^^,^^2^ Persistent changes in calcium regulation and related hormonal changes after kidney donation could place LKDs at risk of increased bone loss in future years.^1^ This information is critical as it informs potential donors of inherent risks of donation and allows for an understanding of the physiological effects of mild reductions in kidney function without the confounding effects of underlying kidney disease. In an editorial accompanying our previous study in *Kidney International,* Evenepoel and Naesens^3^ called for a study of fracture incidence in LKDs.

In our recently published survey-based study,^4^ we showed what Evenepoel and Naesens had suggested: that long-term fracture incidence was increased in the vertebral bodies of LKDs compared with strictly matched controls (standardized incidence ratio for vertebral fractures in donors 1.42; 95% CI 1.05-1.83). However, long-term overall fracture incidence (fractures of all types) was reduced in LKDs versus controls (SIR 0.89; 95% CI, 0.81-0.97). The recently published survey-based study included LKDs from 3 large transplant centers in MN who were invited to complete a survey about their bone health and history of fractures, and a group of matched, population-based non-donor controls who completed the same survey but who did not have a history of comorbidities that would have precluded kidney donation (controls were identified from the Rochester Epidemiology Project).

Of particular note, our recently published survey study was the first of its kind to specifically assess the risk of fractures in LKDs a mean of 25 years after kidney donation and was restricted to LKDs who were 50 years of age or older at the time of survey (ie, donors who had attained an age at which fracture risk was common enough to study). This design was important because the lag time from kidney donation to the realization of changes in skeletal properties and fractures is likely decades.

This study was designed to further explore the *mechanisms* leading to changes in bone health and increased rates of vertebral fractures. These findings extend the understanding of bone health in kidney donors to more than 20 years post-donation and provide evidence of persistent effects of kidney donation on calcium-regulating hormones (and related markers) and on bone formation and resorption markers.

## Materials and methods

### Participants

To increase the likelihood of participation in the mechanistic portion of our study, we recruited LKDs only from among those who had originally donated at the Mayo Clinic Rochester site (from among the 3 sites included in our original survey-based report). Potential LKDs were identified from those persons who had endorsed a question on the fracture risk survey indicating that they would be willing to participate in further assessments of hormonal variables and bone health. Potential controls for the mechanistic portion of the study were recruited specifically for this study and were similarly only recruited from among those who had returned a survey and expressed further interest. For each LKD, a matched control was identified and recruited only after the LKD had successfully completed the extensive bone health clinical visit. This was necessary so that a matched control could be identified of the same sex and similar age (matched by birth date ±5 years) for each donor. Participant characteristics of race, ethnicity, and marital status were self-reported by participants as a part of their complete medical records. Anthropometric characteristics of height, weight, and calculated body mass index (BMI) were measured at the time of the bone health clinical visit completed as part of this study. We collected information on previous exposures to medications known to interfere with Ca, vitamin D, or bone metabolism including glucocorticoids, vitamin D, anti-epileptics, bisphosphonates, estrogen or progesterone, diuretics, and rifampin.

The study was approved by the institutional review board of Mayo Clinic and all participants signed the Authorization to Use and Disclose Protected Health Information (Health Insurance Portability and Accountability Act) form and provided informed consent to be included in this study. All bone health clinical visits were completed at the Clinical Research and Trials Unit (CRTU) of the Mayo Clinic in Rochester, MN.

### Clinical assessment of bone health

Participants were contacted via telephone and invited to undergo an extensive clinical visit to assess bone health. In particular, following an overnight fast, participants reported to the CRTU at 0800 hr for a physical examination and collection of blood used for the measurement of serum electrolytes, calcium, inorganic phosphorus, blood pH, ionized calcium, creatinine, blood urea nitrogen, bone-related hormones, and markers of bone formation and resorption (see below).

After physical examination and blood collection, participants underwent bone mineral density (BMD) imaging of the spine (AP and lateral), hip, and forearm^5^ (Lunar iDXA, software version 15), and high-resolution peripheral quantitative computed tomography (HRpQCT) imaging of the tibia and radius.^5-7^ Specifically, areal BMD was measured by dual energy X-ray absorptiometry (DXA) at the lumbar spine (L_1_–L_4_) for both AP and lateral views; at the femur neck, shaft, and total femur (ie, the hip); and at the forearm for both the radius and ulna. Volumetric density and bone microarchitecture were assessed at the distal radius and tibia by HRpQCT (Xtreme CT, Scanco Medical AG, Brüttisellen, Switzerland) according to the manufacturer’s standard in vivo acquisition protocol.^5-7^ Further analysis of HRpQCT imaging was performed using micro-finite element analysis (FEA) to assess load strength (ie, the simulated force needed to cause a bone to fail). All BMD and HRpQCT measurements were also performed in the CRTU.

### Details of laboratory assays

As part of the clinical visit, we measured several bone-related hormones and markers of bone formation and resorption. Hormonal assays included total 25-hydroxyvitamin D (total 25(OH)D is the sum of 25(OH)D_2_ and 25(OH)D_3_) by LC–tandem mass spectrometry (MS); 1,25-Dihydroxyvitamin D (1,25(OH)_2_D) by immuno-extraction and LC–tandem MS; PTH by a 2-site chemiluminescent immunometric assay on Roche Cobas (Roche Diagnostics); and intact fibroblast growth factor 23 (FGF-23) by an ELISA Kit.^1^^,^^2^^,^^8^^,^^9^

Bone formation markers measured in serum included bone alkaline phosphatase (BAP) by an immunoassay from Metra Biosystems (Mountain View, CA); intact procollagen type 1 N propeptide (P1NP) radioimmunoassay (UniQ™, Orion Diagnostica, Espoo, Finland); and osteocalcin (OC) by a 2-site immunoenzymatic sandwich assay on the Roche Cobas e411 (Roche Diagnostics).^9^

Bone resorption markers measured in serum included N-terminal telopeptide of bone collagen (NTx) by a quantitative competitive-inhibition enzyme-linked immunosorbent assay (Osteomark; Ostex International, Seattle, WA); C-terminal telopeptide of bone collagen (CTx) by a 2-site immunoenzymatic sandwich assay on the Roche Cobas e411 (Roche Diagnostics, Indianapolis, IN); and tartrate resistant acid phosphatase 5B (TRAcP5b) by the BoneTRAP immunoassay (Immunodiagnostic Systems PLC, Boldon, United Kingdom).^9^ All laboratory analyses were completed by Mayo Medical Laboratories or in the Immunochemical Core Laboratory (a part of the CRTU).

### Statistical methods

Demographic and clinical characteristics of LKDs and controls are summarized as means and standard deviations or as counts and frequencies in the table of clinical visit characteristics. By design, matching was implemented to balance the LKD and control groups on age and sex. However, unmatched analyses are performed to maximize sample size due to missingness of bone health markers. LKDs and controls are compared across serum bone biomarkers, imaging-based measurements of BMD, and bone morphometry from HRpQCT using linear models and calculating least-squares means (population marginal means) adjusted for age and sex (to account for any residual imbalance due to missing data and to ensure more accurate standard error calculations) and adjusted for BMI (a potential confounder not in the direct causal pathway between kidney donation and bone health measures, but associated with bone structure). The linear models are not adjusted for variables found to be different between LKDs and controls but that are direct measures of kidney function (eg, estimated glomerular filtration rate [eGFR]). In a set of exploratory mechanistic equilibrium analyses, blood markers in the calcium-regulating pathway—including total 25(OH)D, 1,25(OH)_2_D, PTH, and ionized calcium—were compared one-at-a-time in donors and controls accounting for (ie, balanced on) the other pathway measures using least-squares means. Differences in bone health measures between LKDs and controls are described using both absolute differences and 95% confidence intervals (in the original units of the measurement) and percent differences (and 95% confidence intervals) to highlight the potential clinical significance related to the size and direction of the differences. Statistical tests were performed using SAS version 9.4 and at the conventional 2-tailed alpha level of 0.05.

## Results

We were able to recruit and complete bone health clinical visits for 139 LKDs and 139 matched controls. The demographic characteristics, blood serum measures, and kidney function measures at the time of clinical visit are shown in Table 1.

**Table 1:** Baseline characteristics of controls and LKDs included in study.

	**Controls**	**Donors**	
	**(*N* = 139)**	**(*N* = 139)**	***P*-value for**
**Characteristic**	***N* (%) or Mean (SD)**	***N* (%) or Mean (SD)**	**difference** ^a^
**Sex**			—^b^
Female	78 (56.1)	78 (56.1)	
Male	61 (43.9)	61 (43.9)	
Age at donation, years	—^c^	46.16 (10.46)	—^c^
Years after donation	—^c^	21.89 (8.76)	—^c^
Age at study, years	69.16 (7.73)	68.04 (7.51)	.23
**Race**			.73
White	122 (87.8)	119 (85.6)	
Non-White	3 (2.2)	2 (1.4)	
Unknown/refused	14 (10.1)	18 (12.9)	
**Ethnicity**			.99
Hispanic	2 (1.4)	2 (1.4)	
Non-Hispanic	137 (98.6)	137 (98.6)	
**Marital status**			.34
Married	113 (81.3)	108 (77.7)	
Divorced	11 (7.9)	6 (4.3)	
Widowed	6 (4.3)	10 (7.2)	
Single	8 (5.8)	14 (10.1)	
Unknown	1 (0.7)	1 (0.7)	
**Anthropometrics**			
Height, cm	169.00 (8.79)	169.50 (9.39)	.71
Weight, kg	82.21 (19.27)	87.13 (20.01)	.04
Body mass index, kg/m^2^	28.62 (5.69)	30.21 (5.78)	.02
**Serum blood measures**			
Bicarbonate, mEq/L	26.46 (2.09)	26.27 (2.00)	.45
Chloride, mEq/L	103.50 (3.19)	103.80 (2.46)	.50
Potassium, mEq/L	4.42 (0.38)	4.52 (0.37)	.03
Sodium, mmol/L	140.10 (2.99)	140.30 (2.22)	.72
Inorganic phosphorus, mg/dL	3.34 (0.49)	3.40 (0.51)	.32
Total calcium, mg/dL	9.45 (0.43)	9.51 (0.38)	.18
Ionized calcium, mg/dL^d^	5.04 (0.21)	5.13 (0.20)	<.001
Blood pH^d^	7.41 (0.03)	7.39 (0.03)	<.001
**Kidney function measures**			
Blood Urea Nitrogen, mg/dL	15.60 (4.42)	18.35 (5.58)	<.001
Creatinine, mg/dL	0.87 (0.17)	1.06 (0.30)	<.001
eGFR, mL/min/1.73m^2^	82.89 (12.60)	68.73 (14.58)	<.001

a *P*-values for tests of comparison between controls and donors were performed using t-tests with equal variance for continuous characteristics and using Fisher exact test for categorical variables. Fisher exact test was used because of small cell counts across several of the categorical characteristics.

b Controls and donors were matched exactly on sex, so no statistical test of differences was performed.

c Controls do not have an age at time of donation.

d Ionized calcium and blood pH were not available for 1 control and 8 donors.

By design, the controls and donors were balanced on sex. Controls were slightly older than the LKDs at the time of study (but not statistically different), likely due to the delay in recruitment of controls until after the completion of the extensive bone health clinical visit of the matched donor. No differences were observed by self-reported race, ethnicity, or marital status of controls and donors. LKDs had higher weight than controls (donors mean 87.1 kg vs. controls 82.2 kg, *P* = .04), and had a higher BMI (donors mean 30.2 kg/m^2^ vs. controls 28.6 kg/m^2^, *P* = .02). Serum measures were largely similar in donors and controls except for potassium (donors mean 4.52 mEq/L vs. controls 4.42 mEq/L; *P* = .03), ionized calcium (donors mean 5.13 mg/dL vs. controls 5.04 mg/dL; *P* < .001), and blood pH (donors mean 7.39 vs. controls 7.41; *P* < .001). As expected, blood measures of kidney function and eGFR were significantly decreased in donors versus controls (see Table 1). It is likely that the increase in serum potassium concentrations in LKDs versus controls is due to a reduction in estimated GFR. It not known (outside of this study) whether LKDs have reductions in eGFR 20 years after donation. However, it is known that 36 months after kidney donation iohexol clearance (a measure of glomerular filtration rate) is reduced in LKDs versus matched controls.^1^^,^^2^ Likewise, there is a lack of information on changes in calcium-regulating hormones in LKDs. It is known that 36 months after living kidney donation, 1,25-dihydroxyvitamin D concentrations are diminished and PTH concentrations are increased in LKDs versus controls.^1^

A comparison of calcium-regulating hormones between LKDs and controls is shown in Table 2. We observed elevated total 25(OH)D levels (donors mean 48.44 ng/mL vs. controls = 41.58 ng/mL, percent difference = 16.5%; *P* = .009) and reduced 1,25(OH)_2_D levels (donors mean 33.89 pg/mL vs. controls 38.79 pg/mL, percent difference = -12.6%; *P* < .001) in donors versus controls. Serum PTH and FGF-23 concentrations were slightly higher in donors versus controls, but not statistically different. Of note, PTH values in donors were higher in donors versus controls after correcting for changes in ionized calcium (Table 3). We also observed higher concentrations of most (but not all) bone formation and resorption markers in donors versus controls (BAP, 2.4% increase, *P* = .52; P1NP 13.7% increase, *P* = .04; osteocalcin, 19.4% increase, *P* = .003; NTx 16.8% increase, *P* = .002; CTX 16.1% increase, *P* = .01; Table 2).

**Table 2:** A comparison of bone health markers in controls and LKDs.

	**Controls**		**Donors**			**Difference in donors versus controls**		
**Analyte / measurement**	**N** ^**a**^	**Mean (95% CI)** ^**b**^	**N** ^**a**^	**Mean (95% CI)** ^**b**^		**Absolute (95% CI)** ^**c**^	**Percent (95% CI)** ^**d**^	***P*-value**
**Calcium-regulating blood markers**								
Total 25(OH)D, ng/mL	139	41.58 (38.00 to 45.17)	139	48.44 (44.85 to 52.02)		6.86 (1.76 to 11.96)	16.5% (4.2 to 28.8)	.009
1,25(OH)_2_D, pg/mL	139	38.79 (37.09 to 40.49)	139	33.89 (32.19 to 35.60)		−4.90 (−7.32 to −2.47)	−12.6% (−18.9 to −6.4)	<.001
PTH, pg/mL	139	49.51 (45.78 to 53.24)	139	50.51 (46.78 to 54.25)		1.00 (−4.31 to 6.32)	2.0% (−8.7 to 12.8)	.71
FGF-23, pg/mL	139	45.41 (41.25 to 49.58)	139	47.87 (43.70 to 52.03)		2.46 (−3.47 to 8.38)	5.4% (−7.7 to 18.5)	.42
**Bone formation and resorption markers**								
Bone alkaline phosphatase, μg/L	139	20.77 (19.72 to 21.82)	139	21.27 (20.21 to 22.32)		0.50 (−1.00 to 2.00)	2.4% (−4.8 to 9.6)	.52
P1NP, μg/L	139	47.14 (42.80 to 51.48)	139	53.62 (49.28 to 57.96)		6.48 (0.30 to 12.65)	13.7% (0.6 to 26.8)	.04
Osteocalcin ng/mL	139	21.81 (19.86 to 23.76)	139	26.05 (24.10 to 28.00)		4.24 (1.47 to 7.01)	19.4% (6.7 to 32.1)	.003
NTx, BCE/L	138	18.15 (16.79 to 19.50)	139	21.19 (19.84 to 22.54)		3.04 (1.11 to 4.97)	16.8% (6.1 to 27.4)	.002
CTx, ng/mL	139	0.43 (0.39 to 0.47)	139	0.50 (0.46 to 0.54)		0.07 (0.01 to 0.12)	16.1% (3.2 to 29.0)	.01
TRAcP5	139	2.32 (2.22 to 2.42)	139	2.40 (2.30 to 2.50)		0.08 (−0.06 to 0.23)	3.5% (−2.7 to 9.8)	.27
**Bone mineral density (areal)**								
AP spine, g/cm^2^	137	1.20 (1.17 to 1.23)	133	1.25 (1.22 to 1.29)		0.05 (0.01 to 0.10)	4.5% (0.5 to 8.6)	.03
Trabecular bone score^e^	137	1.40 (1.39 to 1.42)	133	1.39 (1.38 to 1.41)		−0.01 (−0.04 to 0.01)	−0.8% (−2.6 to 1.0)	.37
Lateral spine, g/cm^2^	137	0.69 (0.66 to 0.72)	134	0.70 (0.67 to 0.74)		0.01 (−0.03 to 0.06)	2.1% (−4.8 to 9.1)	.54
Femur neck, g/cm^2^	136	0.88 (0.86 to 0.90)	136	0.90 (0.88 to 0.92)		0.02 (−0.01 to 0.05)	2.2% (−0.9 to 5.3)	.17
Femur shaft, g/cm^2^	136	1.13 (1.11 to 1.16)	136	1.15 (1.13 to 1.18)		0.02 (−0.02 to 0.06)	1.8% (−1.3 to 5.0)	.26
Femur total (hip), g/cm^2^	136	0.96 (0.94 to 0.98)	136	0.98 (0.96 to 1.00)		0.02 (−0.01 to 0.05)	2.0% (−1.1 to 5.0)	.20
Radius total, g/cm^2^	139	0.65 (0.64 to 0.66)	135	0.66 (0.64 to 0.67)		0.01 (−0.01 to 0.03)	1.2% (−1.9 to 4.4)	.44
Ulna total, g/cm^2^	139	0.62 (0.60 to 0.63)	135	0.64 (0.62 to 0.65)		0.02 (0.00 to 0.04)	2.7% (−0.7 to 6.1)	.12
Skin AGEs^f^	139	2.55 (2.45 to 2.64)	136	2.68 (2.58 to 2.77)		0.13 (−0.01 to 0.27)	5.1% (−0.3 to 10.4)	.06
**Bone microstructure and strength (from HRpQCT of the tibia)**								
BV/TV fraction	136	0.24 (0.23 to 0.25)	134	0.23 (0.23 to 0.24)		−0.01 (−0.02 to 0.00)	−3.1% (−8.2 to 2.0)	.24
Trabecular number, 1/mm	136	1.48 (1.44 to 1.53)	134	1.44 (1.40 to 1.49)		−0.04 (−0.11 to 0.03)	−2.7% (−7.2 to 1.8)	.24
Trabecular thickness, mm	136	0.27 (0.26 to 0.27)	134	0.26 (0.26 to 0.27)		0.00 (−0.01 to 0.00)	−1.1% (−3.1 to 0.9)	.28
Trabecular separation, mm	136	0.72 (0.68 to 0.75)	134	0.75 (0.71 to 0.78)		0.03 (−0.02 to 0.08)	4.3% (−2.9 to 11.5)	.24
Cortical thickness, mm	133	2.35 (2.29 to 2.42)	137	2.30 (2.23 to 2.37)		−0.06 (−0.16 to 0.04)	−2.4% (−6.6 to 1.7)	.25
Cortical area, mm^2^	133	176 (172 to 180)	137	171 (167 to 175)		−5 (−11 to 1)	−2.8% (−6.0 to 0.5)	.09
μFEA failure load, N	135	9985 (9674 to 10 296)	133	9618 (9304 to 9932)		−367 (−813 to 79)	−3.7% (−8.1 to 0.8)	.11

a Number of controls and donors included in comparison. Not all measurements were available for all controls and donors.

b Population marginal means (also called least-squares means) in controls and donors are adjusted for (ie, balanced on) age, sex, and BMI.

c Absolute differences are calculated in the original units and reflect the difference in donors as compared with controls and adjusted for age, sex, and BMI.

d Percent differences are calculated as the value in donors minus the value in controls divided by the value in controls.

e Trabecular bone score is measured in the AP spine on DXA and is positively correlated with number of trabeculae. It is a general measure of the structural microarchitecture of trabecular bone but from 2-dimensional imaging (lower values are worse).

f Skin advanced glycation end-products (AGEs) were measured using autofluorescence and are associated with biological aging and inflammation (higher values are worse).

**Table 3:** A comparison of calcium-regulation pathway blood serum markers in controls and LKDs using exploratory mechanistic equilibrium analyses (see Materials and methods).

	**Controls** ^**a**^	**Donors** ^**a**^	**Difference in donors versus controls**		
**Analyte**	**Mean (95% CI)** ^**b**^	**Mean (95% CI)** ^**b**^	**Absolute (95% CI)** ^**c**^	**Percent (95% CI)** ^**d**^	***P*-value**
Total 25(OH)D, ng/mL	41.85 (38.21 to 45.49)	48.21 (44.47 to 51.95)	6.37 (0.96 to 11.77)	15.2% (2.3 to 28.1)	.02
1,25(OH)_2_D, pg/mL	39.13 (37.43 to 40.82)	33.68 (31.94 to 35.43)	−5.44 (−7.93 to −2.95)	−13.9% (−20.3 to −7.5)	<.001
PTH, pg/mL	46.89 (43.24 to 50.54)	52.98 (49.22 to 56.73)	6.08 (0.66 to 11.51)	13.0% (1.4 to 24.5)	.03
Ionized calcium, mg/dL	5.04 (5.00 to 5.07)	5.13 (5.09 to 5.16)	0.09 (0.04 to 0.14)	1.8% (0.8 to 2.8)	<.001

a Analyses were performed on the 138 controls and 131 donors for whom all 4 serum markers were available.

b Each row in the table summarizes the population marginal means (also called least-squares means) in controls and donors after adjustment for (ie, balancing on) age, BMI, and each of the markers in the calcium-regulation pathway other than the one being compared. For example, the difference in donors versus controls for total 25(OH)D is calculated at the mean value of age, BMI, 1,25(OH)_2_D, PTH, and ionized calcium (the mean in donors and controls pooled). In effect, least-squares means compare each analyte in donors and controls by “loading” or “concentrating” all of the calcium-regulation pathway dysfunction into each pathway node, one-at-a-time.

c Absolute differences are calculated in the original units of the measurement and reflect the difference in donors as compared with controls.

d Percent differences are calculated as the value in donors minus the value in controls divided by the value in controls.

BMD measures were similar in donors and controls after adjustment for age, sex, and BMI except for a higher BMD at the AP spine in donors. We further investigated whether LKDs may have deficits in bone microstructure and strength from HRpQCT measures at the tibia. However, none of the HRpQCT-based measures of trabecular and cortical bone at the tibia were statistically significantly different in donors versus controls (Table 2; findings were similar at the radius, not shown). Results trended toward decreases in donors versus controls for several measures of bone microstructure including trabecular bone volume fraction (BV/TV), trabecular number, trabecular thickness, cortical thickness, and cortical area. Trabecular separation (ie, the diameter of the cavities in trabecular bone) trended toward increases in donors and was consistent with decreases in the other measures. The data, although internally consistent, were statistically nonsignificant. Micro- FEA similarly trended toward lower failure loads in donors versus controls (donors mean 9618 N vs. controls 9985 N, percent difference = -3.7%; *P* = .11, statistically nonsignificant). Of interest, we also found a suggestive increase in skin advanced glycation end-products (AGEs) in LKDs compared with controls.

**Figure 1:**
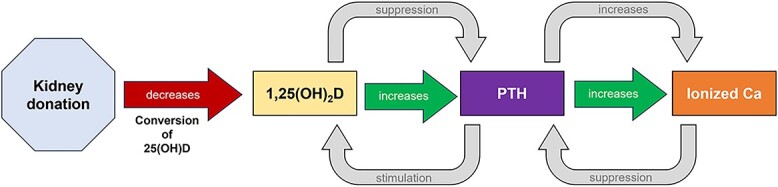
Calcium-regulation pathway and its relation to kidney donation. The hypothesized cascade of events leading to dysfunction in the calcium-regulation pathway. Kidney donation (via decreased renal mass) decreases the ability to convert 25(OH)D to biologically active 1,25(OH)_2_D. Less 25(OH)D converted in the kidneys to 1,25(OH)_2_D leads to observed increases of serum 25(OH)D in donors. A decrease in circulating 1,25(OH)_2_D leads to an increase in PTH (via reduced suppression), and further increases in ionized calcium (via positive feedback).

In further exploratory mechanistic equilibrium analyses, we performed a set of adjusted (balanced) analyses of serum measurements in the calcium-regulating pathway—namely total 25(OH)D, 1,25(OH)_2_D, PTH, and ionized calcium (Figure 1). While PTH was not statistically different in donors versus controls in overall analyses, more nuanced mechanistic analyses demonstrated that PTH is indeed elevated in donors versus controls when balancing on the other calcium-regulating components (Table 3). These analyses unmask a persistent (ie, more than 20 years after donation) dysfunction of the calcium-regulation pathway in donors that manifests clinically as increases in 25(OH)D, decreases in 1,25(OH)_2_D, increases in PTH (largely masked), and increases in ionized calcium (see Table 3).

## Discussion

As expected, blood measures of kidney function and eGFR were significantly decreased in donors versus controls. We also observed decreased 1,25(OH)_2_D concentrations and increased 25(OH)D concentrations in donors versus controls. Less expected, we found increases in potassium, increases in ionized calcium, and decreases in blood pH (ie, more acidic) in donors versus controls, which informed further exploratory analyses of the calcium-regulation pathway.

Serum PTH and FGF-23 concentrations were slightly increased in donors versus controls, but not significantly so. We hypothesize that the primary driver of secondary hyperparathyroidism is a lower 1,25-dihydroxyvitamin D serum concentration, which is associated with a reduction in intestinal calcium transport and negative calcium balance.^10^^,^^11^ The lack of significant differences in measurable PTH between donors and controls is likely due to the increase in ionized calcium levels that occurs on account of acidemia and a reduced blood pH.^12^^,^^13^ Indeed, our mechanistic exploratory analyses suggest that the dysfunction in the calcium-regulation pathway manifests in clinically measurable differences in 1,25(OH)_2_D and ionized calcium, whereas PTH levels seem to maintain equilibrium. We acknowledge that part of the difficulty in detecting clinically measurable differences in PTH levels may be related to the higher between-person variability of this clinical measurement compared with others. It is also difficult to capture temporal fluctuations in PTH levels in a cross-sectional study, where all serum values of participants were measured at one point in time.

Persistent dysfunction in the calcium-regulation pathway, particularly increases in PTH, may predominantly cause deficits in cortical bone.^14^ We posit that the reductions in 1,25(OH)_2_D concentrations and increases in ionized calcium observed in the donors leads to some degree of secondary hyperparathyroidism, increases in bone turnover, and cortical thinning. We did observe higher concentrations of bone formation and resorption markers in donors versus controls, most notably in P1NP, osteocalcin, NTx, and CTx. All of these bone turnover markers are consistent with increased rates of bone loss and possible long-term decreases in bone density and increases in fracture risk. Despite observing an overall decrease in all types of fractures in LKDs versus controls, we observed an increase in vertebral fractures in LKDs versus controls.^4^ This observation suggests differing mechanisms of bone remodeling at the spine (vertebra) versus the appendicular skeleton. As shown in Table 2, micro-FEA failure load and cortical area were lower (though not statistically significant) in LKDs versus controls at the tibia. We note that vertebrae similarly have a thin cortical shell,^15^ and further thinning of this cortical shell could explain the increase in fractures specifically of the vertebrae, as has been observed in population studies of persons with primary hyperparathyroidism.^16^

Considering the increase in vertebral fractures in donors versus controls, the increases in BMD at the AP spine are somewhat surprising, but may be due to confounders (eg, the AP spine can be artifactually elevated due to osteoarthritis, which may be more common in donors for unclear reasons). We also note that another study found similar (and somewhat counter-intuitive) BMD increases in persons with type 2 diabetes versus non-diabetics.^17^

Finally, we found that LKDs tended to have higher skin AGEs, which have been used as a biological marker of aging and overall mortality.^18^ In a previous study, it was also found that higher skin AGEs were correlated with impaired bone material properties (ie, “bone quality”) as assessed by microindentation.^17^ Thus, another possibility for the observed increase in vertebral fracture risk in LKDs may be impaired bone quality related to an accelerated aging phenotype secondary to a reduction in eGFR.

Our study has many strengths. First, we were able to recruit LKDs at a mean of more than 20 years after kidney donation and corresponding matched controls from a related survey-based study of fractures. To our knowledge, this is the first study of its kind to demonstrate persistent and long-term changes of calcium-regulation and bone-turnover markers in LKDs. Second, in addition to serum markers of bone health, we were able to include bone imaging of LKDs and controls. While the imaging findings were not statistically significantly different between LKDs and controls, the direction of findings (particularly the bone microstructure findings from HRpQCT) are consistent with and strengthen both the mechanistic findings of dysfunction in the calcium-regulation pathway and the survey-based findings of higher rates of vertebral fractures in LKDs versus controls.

This study also has several limitations. Donors and controls who participated in the study were volunteers and may have differed from the group of all LKDs and the population at large. However, for the purpose of a mechanistic blood marker and imaging-based study, LKDs and controls were similar on age and sex (by design) and in several other self-reported socio-demographic characteristics (race, ethnicity, and marital status). Reassuringly, our results confirmed that kidney donors had worsened measures of kidney function. More interestingly, we found persistent and long-term altered calcium-regulating hormones and altered bone turnover markers in LKDs. Another limitation of our findings is that the self-reported race and ethnicity of LKDs and controls was less diverse than some other populations in the United States and worldwide. It is likely that our study is under-powered for the detection of changes in bone structure.

In summary, our data show persistent reductions in 1,25-dihydroxyvitamin D in older LKDs at a mean of more than 20 years after kidney donation. This reduction is almost certainly due to a reduction in renal mass. We also observed reductions in blood pH in LKDs suggesting the presence of abnormalities in hydrogen ion excretion, and similar subtle abnormalities in potassium excretion in LKDs. The change in blood pH is associated with an increase in the serum ionized calcium which may mask the increase in PTH that may have been otherwise clinically measurable. Furthermore, our novel exploratory mechanistic analyses suggest that persistent dysfunction in the calcium-regulation pathway (including elevations in PTH) and accompanying increases in bone formation and resorption markers may drive long-term decreased bone health in donors versus controls. The changes in calcium-regulating hormones in LKDs are also associated with suggestive and consistent, but statistically nonsignificant, changes in bone properties including decreases in cortical thickness, decreases in failure load, and possible decreases in bone material properties (ie, “bone quality”) related to increases in AGEs. Treatment options to increase biologically available 1,25 dihydroxyvitamin D by making more substrate available (25-hydroxyvitamin D) may be helpful in improving long-term bone quality in LKDs, and merits further study.

## Author contributions

Brandon R. Grossardt (Conceptualization, Data curation, Formal analysis, Investigation, Resources, Validation, Visualization, Writing—original draft, Writing—review & editing), Hilal Maradit Kremers (Conceptualization, Data curation, Formal analysis, Investigation, Writing—review & editing), Adam Miller (Conceptualization, Data curation, Formal analysis, Investigation, Supervision, Writing—review & editing), Bertram Kasiske (Conceptualization, Data curation, Formal analysis, Funding acquisition, Investigation, Writing—review & editing), Arthur Matas (Conceptualization, Data curation, Formal analysis, Funding acquisition, Investigation, Writing—review & editing), Sundeep Khosla (Conceptualization, Data curation, Formal analysis, Investigation, Writing—review & editing), Walter K. Kremers (Conceptualization, Data curation, Formal analysis, Investigation, Methodology, Writing—review & editing), Hatem Amer (Conceptualization, Data curation, Formal analysis, Investigation, Methodology, Writing—review & editing), and Rajiv Kumar (Conceptualization, Data curation, Formal analysis, Funding acquisition, Investigation, Methodology, Project administration, Resources, Software, Supervision, Validation, Visualization, Writing—original draft, Writing—review & editing)

## Funding

This study was supported directly by grant funding to R.K. from the National Institutes of Health (grant R01 DK125252). This study used the resources of the Rochester Epidemiology Project (REP) medical records-linkage system, which was supported by the National Institute on Aging (R33 AG058738), by the Mayo Clinic Research Committee, and by fees paid annually by REP users. The funding sources had no role in the design and conduct of the study; the collection, management, analysis, and interpretation of the data; the preparation, review, or approval of the manuscript; and the decision to submit the manuscript for publication. The content of this article is solely the responsibility of the authors and does not necessarily represent the official views of the National Institutes of Health or the affiliated institutions of the authors.

## Conflicts of interest

R.K. reported receiving grants from Mayo Clinic Rochester during the conduct of the study and nonfinancial support from Bridge Bio outside the submitted work. No other disclosures were reported. The current clinical investigation is an original full-length submission.

## Data availability

The individual-level data on which this article is based are not made publicly available to ensure the privacy of the study participants. Data may be obtained by contacting the corresponding author and by including a brief outline (no longer than one page) of the intended use. Release of so-called “de-identified” individual-level data may require a signed data sharing agreement by those requesting access.

## Use of AI technologies

No generative AI and AI-assisted technologies were used in in the writing process.
